# Effects of deep slow-release fertilizer on melon yield, economic benefits and rhizosphere microorganisms

**DOI:** 10.3389/fpls.2026.1831940

**Published:** 2026-06-03

**Authors:** Miao Yan, Qigan Liang, Juntao Yang, Jiancai Mao, Qiang Wang, Tao Xiong, Guozhi Hu

**Affiliations:** 1 Institute of Fruits and Vegetables, Xinjiang Academy of Agricultural Sciences, Urumqi, China; 2 Hainan Sanya Breeding Center of Xinjiang Uygur Autonomous Region Academy of Agricultural Sciences, Sanya, China

**Keywords:** deep application, slow-release fertilizer, soil microorganisms, economic benefits, yield

## Abstract

Simplified fertilization is a core technology for achieving efficient melon cultivation. However, the optimal application depth of slow-release fertilizers in the major melon-producing regions of Xinjiang remains poorly defined. In this study, field experiments were conducted to evaluate three application depths of slow-release fertilizer: 15 cm (SF15), 25 cm (SF25), and 35 cm (SF35). Conventional fertilization (CF; 20–25 cm) and a no-fertilizer treatment (F0) were included as controls to systematically investigate the effects of application depth on nutrient accumulation, yield, economic benefits and rhizosphere microbial communities of melon. The results showed that CF treatment primarily increased soil potassium content during the middle and late growth stages and promoted potassium accumulation in fruits, while enriching beneficial microbial taxa associated with carbon cycling and plant stress resistance. In contrast, the SF35 treatment significantly increased soil nitrogen and phosphorus contents and their accumulation in fruits during the same growth stages. This treatment also enriched nitrogen-cycling bacteria and phosphorus solubilizing fungus. In addition, SF35 consistently increased the ACE index of rhizosphere bacterial communities throughout the growth period and adjusted soil pH to a range more suitable for melon growth (6.07–6.62). Importantly, melon yield under SF35 were maintained at levels comparable to those under CF, while reducing labor costs and improving economic benefits. In conclusion, 35 cm is recommended as the optimal application depth of slow-release fertilizer for melon in Xinjiang. This practice enhances key functional microbial groups and soil physicochemical properties, while achieving yield and quality comparable to those of CF in a single application. The findings of this study can provide scientific support for the standardized application of simplified fertilization technology in Xinjiang melon production.

## Introduction

Melon (*Cucumis melo* L.) is an important horticultural crop worldwide because of its high nutritional and economic value and strong consumer demand. According to recent statistics, the global cultivation area of melon is approximately 1.72 million hectares, with an annual production exceeding 40 million tons and an export value of approximately USD 2.4 billion (Mahmoodi et al., 2020). Xinjiang, China, represents one of the major production regions for high-quality melon cultivation, owing to its unique climatic conditions, including large diurnal temperature fluctuations, abundant solar radiation, and low precipitation. The region ranks among the leading melon-producing areas in China in both cultivation area and yield and has become an important pillar of local agricultural development and rural income generation (Wang et al., 2022; Zhao et al., 2025). However, due to its vast territory and low population density, Xinjiang faces severe labor shortages in agricultural production. The region’s large-scale melon industry urgently calls for labor-saving, cost-effective, and convenient simplified cultivation technologies, which are of great practical significance for promoting the green and efficient development of Xinjiang’s melon industry.

In current production systems, melon fertilization relies predominantly on basal fertilizer application, supplemented by multiple topdressing applications during the growing season, a practice similar to that used for most field crops (Zhou et al., 2022). However, plants typically absorb only 50-60% of applied nitrogen and potassium and merely 10-25% of applied phosphorus (Bi et al., 2020). Such conventional fertilization practices lead to a range of adverse consequences, including water pollution, increased greenhouse gas emissions, and substantial waste of fertilizer resources (Lin et al., 2019; Beig et al., 2020). Moreover, the reliance on repeated topdressing not only increases labor input but also reduces management efficiency in large-scale cultivation systems due to frequent field operations, thereby intensifying labor inefficiency (Wang et al., 2021c). Excessive and unreasonable fertilization, driven by compensatory applications in response to nutrient losses, has consequently become a major contributor to agricultural non-point source pollution in China (Li et al., 2013). Therefore, there is an urgent need to adopt scientifically optimized fertilization strategies, including improving application methods, reducing material inputs, and enhancing fertilizer use efficiency.

Slow-release fertilizers are a new class of environmentally friendly compound fertilizers that release nutrients gradually. They are characterized by a prolonged release period and a sustained, slow nutrient release rate, enabling crops to meet their nutrient requirements at different growth stages from a single application (Shen et al., 2021; Eddarai et al., 2024). Research on slow-release fertilizers has primarily focused on improving coating materials (Wang et al., 2022; Alikhani et al., 2023), combining them with other fertilizers (Liang et al., 2022; Hu et al., 2023), and optimizing reduced application rates (Xu et al., 2026). However, the nutrient release rate is also influenced by the placement and soil environment of the fertilizer (Wang et al., 2021a). Previous studies demonstrated that the combined application of slow-release nitrogen fertilizer and urea under subsoiling tillage can effectively regulate nitrogen uptake and transport, thereby improving water and nitrogen use efficiency and stabilizing maize yield (Fan et al., 2025). Similarly, in rice, the combined use of slow-release nitrogen fertilizer with side-depth urea application has been shown to significantly increase grain yield and nitrogen use efficiency by optimizing dry matter accumulation and nitrogen transport (Hu et al., 2025). In contrast, deep application of slow-release fertilizer has been reported to exert only limited effects on potato tuber yield (Niedziński et al., 2024). These findings indicate that crop responses to the deep application of slow-release fertilizers vary considerably among species. Therefore, systematic and crop-specific studies are urgently needed to determine the optimal fertilization depth for melon cultivation.

Soil microorganisms are the most active and functionally important components of the soil ecosystem, playing critical roles in the decomposition, formation, nutrient cycling, and energy flow of soil organic matter (Zhang et al., 2025). A diverse and abundant microbial community is essential for maintaining ecosystem functions and services (Fonturbel et al., 2012; Navarro-Noya et al., 2013). However, soil microbial communities are highly sensitive to environmental factors, including fertilizer types and fertilization methods (Zhao et al., 2014; Huang et al., 2020). For instance, compared with conventional urea, slow-release fertilizers have the potential to enhance soil bacterial diversity and community structure (Meng et al., 2024a). Similarly, the application of organic fertilizer derived from bitter melon has been shown to modify the composition and function of bacterial and fungal communities in the melon rhizosphere, promote the growth of beneficial microbes, and increase fruit sugar content (Hua et al., 2023). Short-term deep application of nitrogen fertilizer has been shown to regulate soil nitrogen status and reshape both dominant and rare bacterial communities, thereby optimizing species assembly patterns and co-occurrence networks within the rhizosphere microbiome and contributing to the stability of the rhizosphere microecological environment (Li et al., 2021b). In addition, deep application of compost can selectively enrich specific microbial communities in the pepper rhizosphere, including functional taxa involved in nitrogen cycling, such as *Azoarcus* and *Alcaligenes*, which possess both heterotrophic nitrification and denitrification capacities (Córdoba-Agudelo et al., 2026). Despite these advances, little is known about the response of rhizosphere microbial communities to the application of slow-release fertilizers in melon cultivation.

Investigating changes in the rhizosphere microbial community under slow-release fertilization is therefore essential for understanding the microbial ecosystem throughout the melon growth cycle. Furthermore, it remains to be verified whether one-time simplified fertilization can reduce labor input while maintaining stable yield. Accordingly, this study systematically investigated the effects of deep application of slow-release fertilizer on melon growth, yield, economic benefits and rhizosphere soil microbial communities, aiming to clarify the feasibility and optimal parameters of this technique, and provide theoretical support for establishing labor-saving, efficient and green fertilization systems in arid regions.

## Methods

The experiment was conducted at the Hami melon test base in Yarkun Town, Gaocun District, Turpan, Xinjiang, China (42°13′N, 89°15′E), which is an arid region. The mean annual precipitation ranges from 14 to 17 mm, and the mean annual temperature is 15.70°C. The extreme high temperature in summer is 49.0°C. Daily mean temperature and precipitation during the melon growing season in 2025 were obtained from the China Meteorological Network and are presented in **Supplementary Figure 1**. The soil at the site is classified as cultivated brown desert soil. The main physicochemical properties of the soil were as follows: organic matter, 10.35 g kg⁻¹; total nitrogen, 0.76 g kg⁻¹; total phosphorus, 1.03 g kg⁻¹; total potassium, 13.17 g kg⁻¹; water-soluble nitrogen, 95.42 mg kg⁻¹; available phosphorus, 32.48 mg kg⁻¹; and available potassium, 364.3 mg kg⁻¹.

### Experimental design

The experiment was arranged in a single-factor randomized block design with five treatments and three replicates. Each plot covered an area of 20 m² (5 m × 4 m), and buffer rows were established between plots to prevent fertilizer movement and runoff. The treatments consisted of four fertilization regimes and a control: slow-release fertilizer applied at depths of 15 cm (SF15), 25 cm (SF25), and 35 cm (SF35); a conventional fertilization treatment (CF); and a no-fertilizer control (F0). For the CF treatment, fertilizers were applied at a depth of 20–25 cm. The total fertilizer application rate was identical across all fertilized treatments, supplying 210 kg N hm^-2^, 180 kg P_2_O_5_; hm^-2^, and 150 kg K_2_O hm^-2^. The melon-specific slow-release fertilizer containing 0.51% nitrification and urease inhibitors, with an N-P_2_O_5_-K_2_O ratio of 21%-14%-16%, was applied once as basal fertilizer before sowing using mechanical placement. In the CF treatment, nitrogen fertilizer was supplied as urea (46% N), with 20% applied as basal fertilizer beneath the plastic mulch along the ridge and the remaining 80% applied via fertigation at the five-leaf stage, flowering and fruiting stage, and fruit enlargement stage, at rates of 25%, 25%, and 30%, respectively. Phosphorus fertilizer was applied as superphosphate (46% P_2_O_5_;), with 100% applied as basal fertilizer beneath the ridge mulch. Potassium fertilizer was supplied as potassium sulfate (50% K_2_O), with 50% applied as basal fertilizer and the remaining 50% applied at the fruit enlargement stage. All treatments were irrigated with a total of 3600 m^3^·hm^-2^ using mulched drip irrigation (Xinjiang Administration for Market Regulation, 2019). The widely cultivated melon ‘Xizhoumi No. 25’ was provided by the Grape and Fruit Research Institute of Xinjiang Uygur Autonomous Region as the experimental material. Melons were sown on 5 April and harvested on 30 June. Plants were grown in a single-row system with main vine pruning, a row spacing of 2 m, and a plant spacing of 0.8 m. All treatments were managed uniformly according to local standard agronomic practices.

### Measurement of soil-related indicators

At the flowering and fruiting stage (4 days after flowering), fruit enlargement stage (18 days after flowering), and maturity stage (40 days after flowering), rhizosphere soil samples were collected from each treatment. Thirty melon plants with uniform growth and no visible pests or diseases were selected using an “S-shaped” sampling pattern. Plants were carefully excavated, and loosely attached soil was gently shaken off. The soil tightly adhering to the root surface (approximately 1–2 mm thick) was collected as rhizosphere soil (Luo et al., 2025). The collected soil samples were thoroughly homogenized and divided into two subsamples.

One subsample was rapidly frozen in liquid nitrogen and stored at -80 °C for high-throughput sequencing, with 4 biological replicates per treatment. Total microbial genomic DNA was extracted using the Power Soil^®^ DNA Isolation kit (MOBIO, Carlsbad, CA, USA) according to the manufacturer’s instructions. DNA integrity and fragment size were assessed by electrophoresis on a 1% agarose gel, and DNA samples were stored at −20 °C until further analysis. The ITS1 region of soil fungi was amplified using the primers ITS1F and ITS2 (2043R), while the V3–V4 region of the bacterial 16S rRNA gene was amplified using primers 338F and 806R. PCR products were separated on a 2% agarose gel and purified using the Agencourt AMPure XP nucleic acid purification Kit (Beckman Coulter, Brea, CA, USA). After quality verification, the purified amplicons were submitted to Metware Biotechnology Co., Ltd (Wuhan, China) for library preparation and sequencing. Amplicon sequencing was performed using the Illumina MiSeqTM platform. Raw reads were deposited in the NCBI Sequence Read Archive (SRA) under accession numbers [PRJNA1404602] (bacteria) and [PRJNA1404617] (fungi).

The second subsample was air-dried naturally for the determination of soil physicochemical properties. Soil pH was measured using a HACH HQ30d pH meter (BANTE, Shanghai, China) with a soil-to-water ratio of 1:2.5 (w/v). Soil organic matter content was determined by the potassium dichromate oxidation-reduction titration method. Available nitrogen and total nitrogen contents were determined using the Kjeldahl method. Available phosphorus and total phosphorus contents were determined using the molybdenum-sulfur antimony colorimetric method, while available potassium and total potassium contents were measured by flame photometry (Bao, 2000).

### Determination of nutrient accumulation, yield and fruit quality

At the flowering and fruiting stage, fruit enlargement stage, and maturity stage, samples of aboveground and belowground plant tissues were collected. Five plants were sampled from each treatment, with three biological replicates. Roots, stems, leaves, and fruits were separated and first heated at 105 °C for 30 min to deactivate enzymes, then oven-dried at 75 °C to constant weight. Dry biomass was recorded for each organ. The dried samples were ground and passed through a 0.5 mm sieve for nutrient analysis. Plant samples were digested using an H_2_SO_4_–H_2_O_2_ digestion procedure. Nitrogen content was determined using the Kjeldahl method, phosphorus content by the molybdenum–antimony colorimetric method, and potassium content by flame photometry. Nutrient accumulation was calculated as follows (Ray et al., 2020): Nutrient accumulation (kg·hm^-^²) = nutrient concentration × dry matter mass.

At harvest, ten fruits were randomly collected from each treatment. Individual fruit weight was recorded, and yield per unit area was calculated based on planting density. The vitamin C (VC) content was determined by 2,6-dichlorophenol indophenol titration, organic acid content by NaOH neutralization titration, and the soluble solids content was measured by PAL-1 handheld refractometer (ATAGO Co., LTD., Japan) (Luo et al., 2025).

### Data analysis and processing

Data analysis and graphical visualization were performed using GraphPad Prism (Version 8.0.1). One-way analysis of variance (ANOVA) was conducted, followed by Tukey’s multiple comparison test. Differences were considered significant at *p* ≤ 0.05. All results are presented as the mean± standard error (SE). Differences among treatments were evaluated using SPSS (Version 21.0). Amplicon sequence variant (ASV) richness and α-diversity indices, including Good’s coverage, Chao1, and Shannon indices, were calculated using Mothur (Version 1.22.2). Redundancy analysis (RDA) was conducted to assess relationships between microbial community composition and environmental variables using the vegan package in R. Correlation analyses between microbial community structure, soil physicochemical properties, and yield were performed using the ggcor package. Taxonomic composition and relative abundance at different taxonomic levels were analyzed using QIIME, and microbial community composition plots and α-diversity boxplots were generated using Origin 2021 software.

## Results

### Effects of fertilization methods on nutrient accumulation and fruit quality of melons

**Figure 1:**
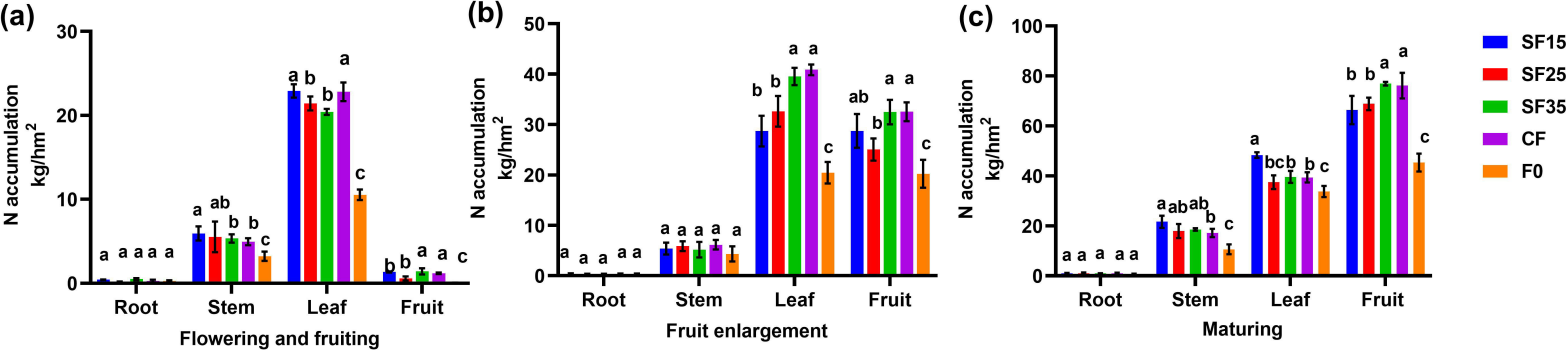
Effects of different fertilization methods on nitrogen accumulation in melon organs. **(a)** Flowering and fruiting stage; **(b)** Fruit enlargement stage; **(c)** maturing stage. SF15, SF25, and SF35 indicate slow-release fertilizer applied at depths of 15, 25, and 35 cm, respectively; CF, conventional fertilization; F0, no fertilizer application. Different letters indicate significant differences among treatments.

Nitrogen accumulation in different melon organs under the various fertilization treatments is shown in **Figure 1**. During the flowering and fruit-setting stage, nitrogen was primarily accumulated in leaves. Nitrogen accumulation in the SF15 (22.91 kg·hm^-^²) and CF (22.81 kg·hm^-^²) treatments was significantly higher than that in other treatments. At the fruit enlargement stage, leaves remained the major site of nitrogen accumulation. The CF (40.84 kg·hm^-^²) and SF35 (39.52 kg·hm^-^²) treatments exhibited significantly greater nitrogen accumulation than the other treatments. Meanwhile, nitrogen accumulation in the fruits increased rapidly, with SF35 (40.84 kg·hm^-^²) and CF (39.52 kg·hm^-^²) showing the highest values. At the maturity stage, fruits became the primary sink for nitrogen accumulation. Under these conditions, the SF35 (76.92 kg·hm^-^²) and CF (76.15 kg·hm^-^²) treatments still exhibited the highest nitrogen accumulation, which was significantly greater than that observed in the other treatments.

**Figure 2:**
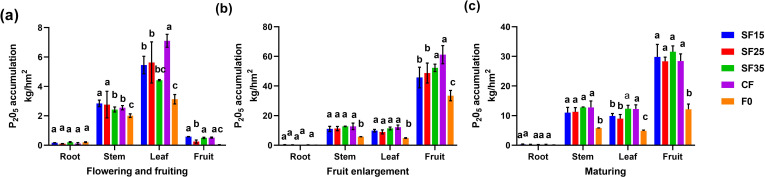
Effects of different fertilization methods on phosphorus accumulation in melon organs. **(a)** Flowering and fruiting stage; **(b)** Fruit enlargement stage; **(c)** maturing stage. SF15, SF25, and SF35 indicate slow-release fertilizer applied at depths of 15, 25, and 35 cm, respectively; CF, conventional fertilization; F0, no fertilizer application. Different letters indicate significant differences among treatments.

Phosphorus accumulation in different melon organs under the various fertilization treatments is shown in **Figure 2**. During the flowering and fruit-setting stage, phosphorus was mainly accumulated in leaves. The CF treatment exhibited the highest phosphorus accumulation in leaves (7.10 kg·hm^-^²), which was significantly greater than that observed in other treatments. At the fruit enlargement stage, the primary site of phosphorus accumulation gradually shifted from leaves to fruits. Phosphorus accumulation in fruits under the CF (61.14 kg·hm^-^²) and SF35 (52.28 kg·hm^-^²) treatments was significantly higher than under other treatments. At the maturity stage, fruits became the main organ for phosphorus accumulation. Although no significant differences were observed among fertilization treatments, the SF35 treatment performed the best (31.57 kg·hm^-^²), which was 11.01% higher than CF.

**Figure 3:**
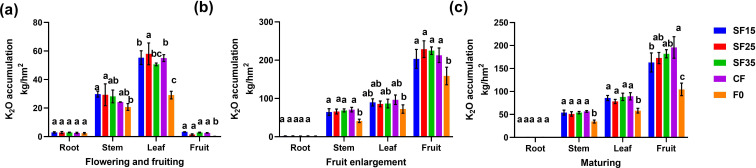
Effects of different fertilization methods on potassium accumulation in melon organs. **(a)** Flowering and fruiting stage; **(b)** Fruit enlargement stage; **(c)** maturing stage. SF15, SF25, and SF35 indicate slow-release fertilizer applied at depths of 15, 25, and 35 cm, respectively; CF, conventional fertilization; F0, no fertilizer application. Different letters indicate significant differences among treatments.

Potassium accumulation in different melon organs under the various fertilization treatments is presented in **Figure 3**. During the flowering and fruit-setting stage, potassium was primarily accumulated in the leaves. The SF25 treatment resulted in the highest potassium accumulation in leaves (57.99 kg·hm^-^²), which was significantly greater than that observed in the other treatments. At the fruit enlargement stage, potassium began to be extensively translocated to the fruits, although no significant differences were detected among the fertilization treatments. At the maturity stage, fruits became the primary organ for potassium accumulation. The CF treatment exhibited the highest potassium accumulation in fruits (285.13 kg·hm^-^²), followed by the SF35 treatment (270.40 kg·hm^-^²), with no significant difference between the two treatments. Overall, the nutrient accumulation patterns under different fertilization treatments indicated that nitrogen is predominantly accumulated in leaves at the flowering, fruit-setting and fruit enlargement stage, then remobilized to fruits at the maturity stage. By contrast, phosphorus and potassium mainly accumulate in leaves during the flowering and fruiting stage and are continuously translocated to fruits in the fruit enlargement and maturity stages. Compared with the CF treatment, the SF35 treatment showed slightly higher nitrogen and phosphorus accumulation, whereas potassium accumulation was slightly lower.

### Effects of fertilization methods on melon yield and fruit quality

As shown in **Table 1**, different fertilization treatments significantly affected melon yield and fruit quality. In terms of yield, the SF35 treatment produced a slightly higher yield than the CF treatment, with an increase of 0.6%. Compared with the F0 treatment, yields under SF35 and CF increased significantly by 45.53% and 44.54%, respectively. Similar trends were observed for single-fruit weight. The fruit appearance of melons under different fertilization treatments is shown as S2. The soluble solid content remained relatively stable among treatments, ranging from 15.05% to 15.62%, with no significant differences detected. The vitamin C content was highest in the CF treatment (297.95 mg/g), which did not differ significantly from that of the SF35 treatment (295.58 mg/g); both values were significantly higher than those observed in the other treatments. Overall, these results indicate that the SF35 treatment achieved yield and quality outcomes comparable to those of conventional fertilization, while exhibiting superior overall performance relative to the SF15 and SF25 treatments. Therefore, SF35 was selected as the representative slow-release fertilizer treatment for subsequent experiments. Together with the no-fertilizer treatment (F0) and the conventional fertilization treatment (CF), it was used for further analyses of soil physicochemical properties and rhizosphere microbial diversity.

**Table 1:** Effects of different fertilization treatments on melon yield and fruit quality.

Treatment	Yield (t/ha)	Longitudinal diameter (cm)	Transverse diameter (cm)	Fruit shape index	Weight per fruit (kg)	Soluble solid (%)	Vitamin C (mg/g)
F0	32.94 ± 1.25 c	14.37 ± 0.27 c	18.15 ± 0.45 b	0.79 ± 0.02 a	1.83 ± 0.07 c	13.13 ± 0.77 a	249.81 ± 3.0 d
SF15	42.66 ± 1.57 b	15.47 ± 0.21 b	21.4 ± 0.70 a	0.73 ± 0.03 a	2.37 ± 0.09 b	15.05 ± 0.87 a	267.56 ± 2.64 c
SF25	41.19 ± 2.13 b	15.43 ± 0.20 b	21.12 ± 0.67 a	0.73 ± 0.02 a	2.29 ± 0.12 b	15.07 ± 0.50 a	289.90 ± 2.66 b
SF35	47.94 ± 1.85 a	16.55 ± 0.25 a	22.90 ± 0.72 a	0.73 ± 0.02 a	2.66 ± 0.10 a	15.62 ± 0.35 a	295.58 ± 2.45 ab
CF	47.61 ± 1.24 a	15.37 ± 0.27 b	21.02 ± 0.48 a	0.73 ± 0.02 a	2.65 ± 0.07 a	15.48 ± 1.01 a	297.95 ± 0.23 a

Different lowercase letters within the same column indicate significant differences among treatments.

### Effects of deep application of slow-release fertilizer on soil physicochemical properties

The effects of different fertilization treatments on soil physicochemical properties during the entire growth phase are shown in **Table 2**. Compared with non-fertilized control (F0) and conventional fertilization (CF), soil pH under the deep application of slow-release fertilizer (SF) treatments remained relatively stable and closer to neutral, ranging from 6.07 to 6.62. In contrast, soils under F0 and CF showed lower pH values (5.85–5.91), indicating slight acidification. Relative to CF, SF significantly increased the total nitrogen and available nitrogen contents during the fruit enlargement and maturation stages, with increases of 51.50% and 3.23% at the enlargement stage and 5.04% and 8.58% at maturity, respectively. Changes in total phosphorus content followed a similar pattern, with SF increasing total phosphorus by 4% and 0.20% at the enlargement and maturity stages, respectively, compared to CF. Moreover, SF significantly increased available phosphorus content at all measured growth stages, with increases of 13.25%, 10.06%, and 14.44% during the flowering and fruiting, fruit enlargement, and maturity stages, respectively. Soil organic matter content was also higher under SF than under CF during the flowering and fruiting and the fruit enlargement stage, with increases of 3.14% and 1.35%, respectively. In contrast, CF resulted in significantly higher available potassium content than SF during the fruit enlargement and maturity stages, with increases of 7.64% and 15.83%, respectively. Overall, slow-release fertilization primarily enhanced soil nitrogen and phosphorus availability during the middle and late growth stages of melon development, whereas conventional fertilization mainly increased soil potassium content.

**Table 2:** Effects of different fertilization treatments on soil physicochemical properties.

Growth stage	Treatment	pH	TN (g/kg)	TP (g/kg)	TK (g/kg)	AN (mg/kg)	AP (mg/kg)	AK (mg/kg)	SOM (g/kg)
Flowing and fruiting	F0	6.56 ± 0.12a	7.48 ± 0.05b	0.17 ± 0.01c	11.76 ± 0.05b	101.62 ± 0.56a	16.79 ± 0.10a	75.72 ± 0.36c	31.08 ± 0.19a
	CF	5.91 ± 0.04b	10.40 ± 0.07a	0.24 ± 0.02a	11.46 ± 0.09c	98.26 ± 0.57b	11.13 ± 0.06c	84.42 ± 0.60b	29.91 ± 0.31b
	SF	6.62 ± 0.23a	6.91 ± 0.06c	0.18 ± 0.01b	12.97 ± 0.05a	81.87 ± 0.38c	14.25 ± 0.14b	104.65 ± 0.70a	30.85 ± 0.21a
Fruit enlargement	F0	7.01 ± 0.05a	7.56 ± 0.14b	0.16 ± 0.14b	9.49 ± 0.03c	89.36 ± 0.56a	9.46 ± 0.06b	45.33 ± 0.38c	31.11 ± 0.14a
	CF	5.85 ± 0.16b	7.34 ± 0.09b	0.25 ± 0.09a	12.97 ± 0.11a	78.51 ± 0.64c	9.91 ± 0.07b	90.00 ± 0.13a	29.57 ± 0.18a
	SF	6.22 ± 0.13a	11.12 ± 0.04a	0.26 ± 0.04a	8.04 ± 0.04b	82.47 ± 0.37b	11.06 ± 0.01a	83.61 ± 0.09b	29.97 ± 0.32a
Maturing	F0	6.89 ± 0.15a	6.71 ± 0.15b	0.18 ± 0.01b	11.76 ± 0.07a	69.92 ± 0.66c	10.54 ± 0.11c	74.64 ± 0.29b	30.46 ± 0.12b
	CF	5.88 ± 0.08b	10.08 ± 0.07a	0.27 ± 0.01a	12.66 ± 0.03a	77.18 ± 0.35b	12.63 ± 0.03b	83.28 ± 0.60a	31.46 ± 0.12a
	SF	6.07 ± 0.11a	10.10 ± 0.10a	0.30 ± 0.01a	12.58 ± 0.11a	83.80 ± 0.33a	15.44 ± 0.03a	71.90 ± 0.31c	29.52 ± 0.25b

TN, total nitrogen; TP, total phosphorus; TK, total potassium; AP, available phosphorus; AK, available potassium. Values are means ± standard error (SE). Different lowercase letters within the same column indicate significant differences among treatments. F0 indicates no fertilization, CF indicates conventional fertilization, and SF indicates deep application of slow-release fertilizer at a depth of 35 cm. The same as below.

### Effects of fertilization treatments on soil microbial α-diversity

**Figure 4:**
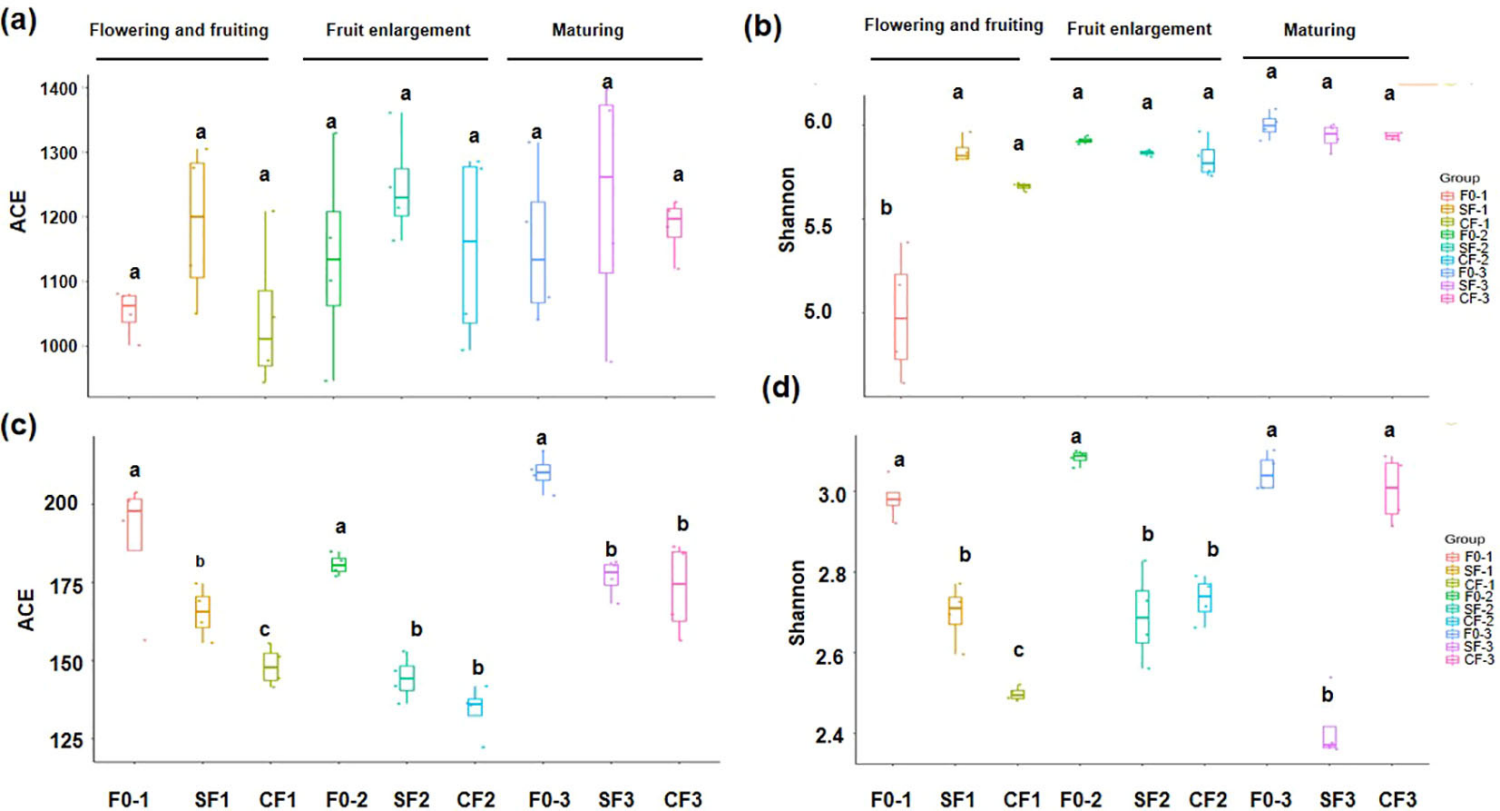
Effects of fertilization treatments on α-diversity of soil bacterial and fungal communities. **(a)** Bacterial ACE index; **(b)** bacterial Shannon index; **(c)** fungal ACE index; **(d)** fungal Shannon index. F0-1, CF1, and SF1 correspond to the flowering and fruiting stage; F0-2, CF2, and SF2 correspond to the fruit enlargement stage; and F0-3, CF3, and SF3 correspond to the maturity stage. Different lowercase letters indicate significant differences among treatments.

To assess the influence of fertilization strategies on rhizosphere microbial communities, bacterial and fungal α-diversity was analyzed at key growth stages of melon development. Compared with CF and F0, SF consistently tended to increase the ACE index of rhizosphere bacterial communities across the entire growth stage of melon. In addition, Shannon index was enhanced under SF at the flowering and fruiting stage (**Figure 4a**). In contrast, fungal communities responded differently to fertilization. Both richness and diversity were consistently lower under fertilization treatments than under F0, as reflected by reduced ACE and Shannon indices (**Figure 4b**). Collectively, the above results demonstrate that SF enhanced rhizosphere bacterial richness across the entire growth stage of melon, while all the fertilization treatments conversely reduced fungal richness and diversity.

### Effects of fertilization treatments on soil microbial ß-diversity

Principal coordinates analysis (PCoA) based on Jaccard distances revealed clear differences in rhizosphere microbial community structure among fertilization treatments. For bacterial communities, the first two principal coordinates explained 32.97% (PCoA1) and 21.70% (PCoA2) of the total variation, accounting for 54.67% cumulatively. For fungal communities, PCoA1 and PCoA2 explained 56.88% and 15.81% of the variation, respectively, with a cumulative contribution of 72.69%. Permutational multivariate analysis of variance (PERMANOVA, Adonis) further confirmed that fertilization treatments significantly affected both bacterial (**Supplementary Figure 3a**) and fungal (**Supplementary Figure 3b**) community structures (*R²* > 0, *P* < 0.05), indicating that between-group variation exceeded within-group variation.

### Effects of fertilization treatments on soil microbial community composition and function

**Figure 5:**
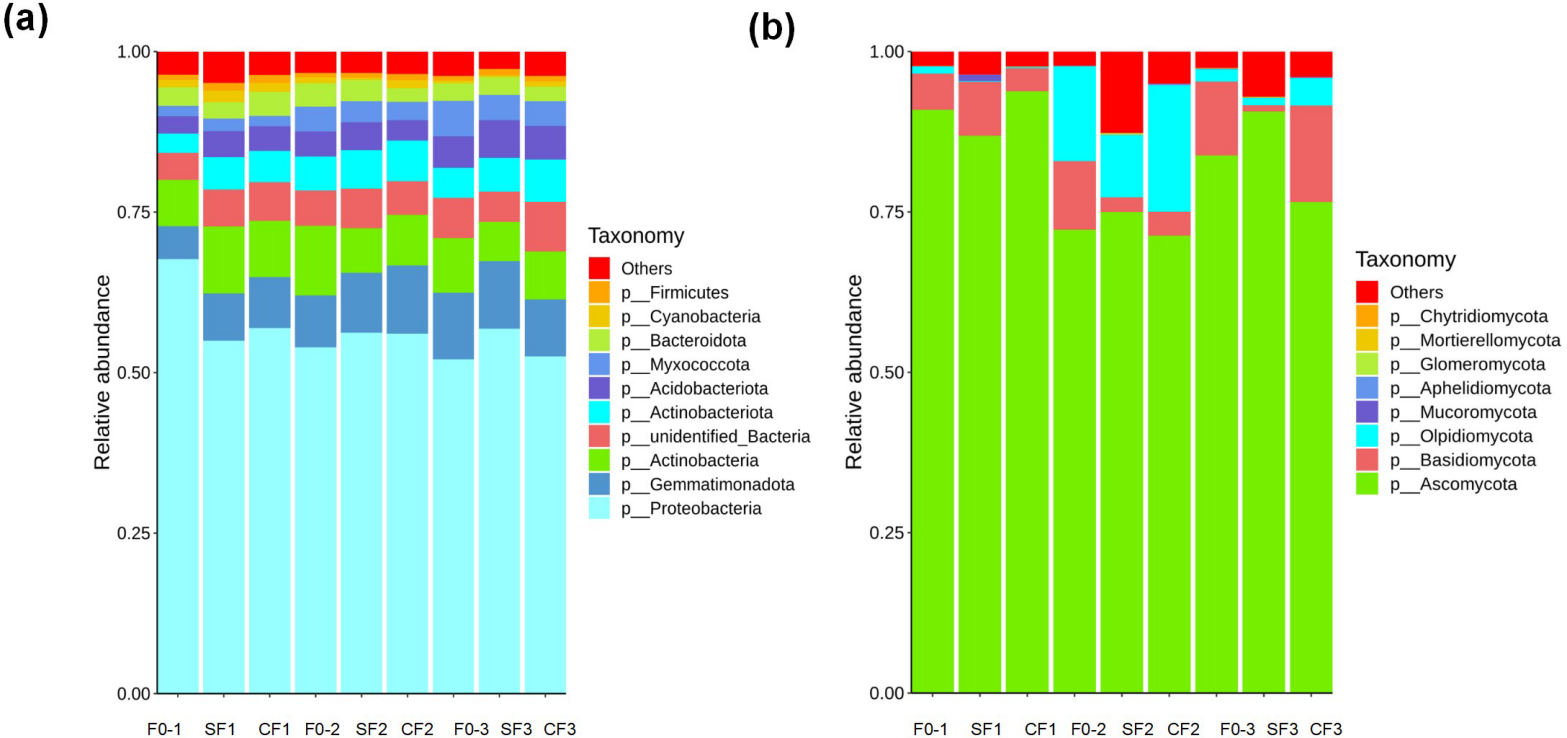
Composition and structure of rhizosphere microbial communities at the phylum level under different fertilization treatments. **(a)** Bacterial phyla; **(b)** fungal phyla. SF indicates deep application of slow-release fertilizer at 35 cm; CF indicates conventional fertilization; F indicates no fertilization. Data are presented for key growth stages: flowering and fruiting (F0-1/CF1/SF1), fruit enlargement (F0-2/CF2/SF2), and maturity (F0-3/CF3/SF3). Relative abundances of dominant phyla are shown.

To investigate how fertilization strategies shape rhizosphere microbial communities, bacterial and fungal composition was analyzed at both phylum and genus levels during the growth phase of melon development. At the phylum level, the bacterial community was dominated by Proteobacteria (52.05%-67.67%), Gemmatimonadota (5.15%-10.65%), Actinobacteria (6.13%-10.84%), Actinobacteriota (2.99%-6.61%), Acidobacteriota (2.71%-5.85%), Myxococcota (1.59%-5.54%), and Bacteroidota (2.10%-3.72%), with Proteobacteria being the most abundant across all treatments (**Figure 5a**). SF treatment promoted a gradual increase in Proteobacteria over the growth phase, while both SF and CF enhanced Proteobacteria abundance relative to F0 in the middle and late stages, with SF > CF (**Figure 5a**). Similarly, SF increased Actinobacteria abundance during the flowering and fruiting stage compared with CF, whereas CF favored Gemmatimonadota early in development (**Figure 5a**). In the fungal community, SF specifically increased Ascomycota in the early stage and consistently reduced Olpidiomycota throughout the growth phase (**Figure 5b**).

**Figure 6:**
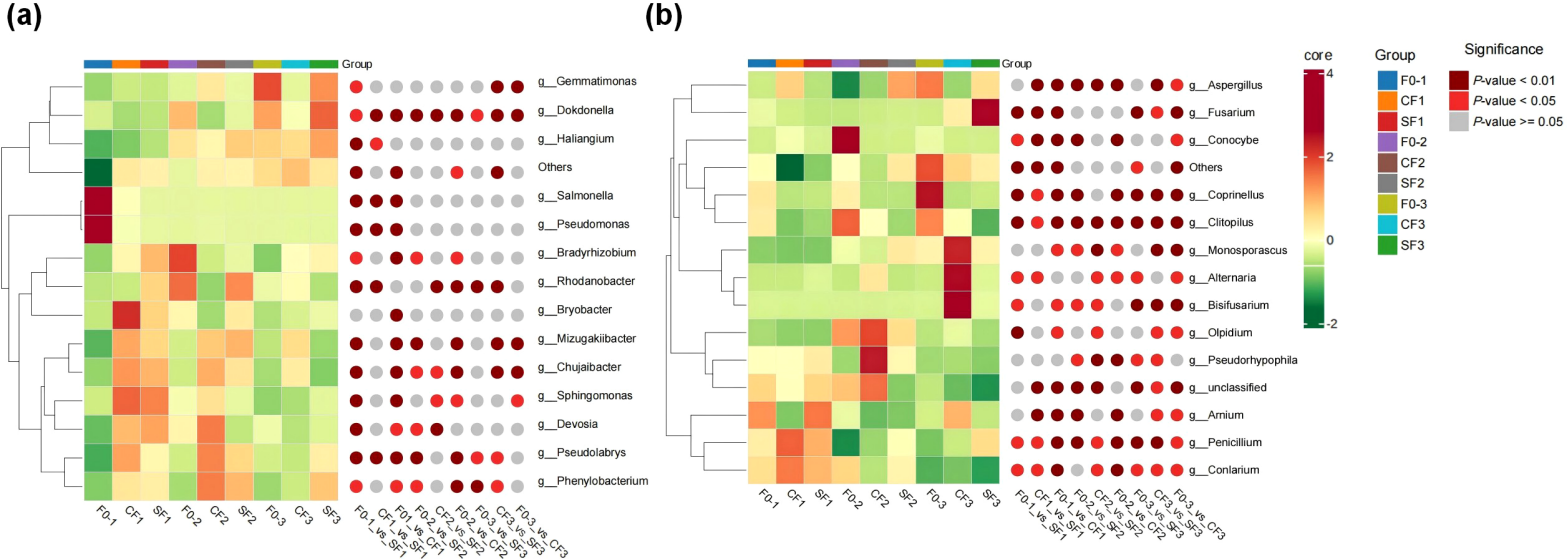
Genus-level composition and structure of rhizosphere microbial communities under different fertilization treatments. **(a)** Bacterial genera; **(b)** fungal genera. Data are presented for key growth stages: flowering and fruiting (F0-1/CF1/SF1), fruit enlargement (F0-2/CF2/SF2), and maturity (F0-3/CF3/SF3). Colored dots denote statistically significant differences among treatments, with dark red indicating *p* < 0.01 and red indicating *p* < 0.05.

At the genus level, dominant bacteria included *Pseudolabrys* (2.40%-5.36%), *Rhodanobacter* (2.35%-5.82%), *Gemmatimonas* (2.19%-5.72%), *Sphingomonas* (2.05%-5.24%), and *Devosia* (1.55% -2.47%). SF markedly increased *Rhodanobacter* during the early and middle stages of fruit development by 58.45% and 3.11-fold, respectively, and slightly enhanced *Devosia* by 2.85%. In contrast, CF favored *Pseudolabrys*, *Gemmatimonas*, and *Sphingomonas* during the early and middle stages, with the increase rates being 21.21% (*P* < 0.05) and 15.92% (*P* > 0.05) for *Pseudolabrys*, 2.82% (*P* > 0.05) and 27.10% (*P* > 0.05) for *Gemmatimonas*, and 5.93% (*P* > 0.05) and 31.41% (*P* < 0.05) for *Sphingomonas*, respectively (**Figure 6a**). For fungi, *Penicillium* (20.22%-53.16%) were dominant. SF significantly increased the abundance of *Penicillium* in the rhizosphere of melon during the middle and late stages of melon fruit development by 17.57% and 35.24%, while the abundance of *Olpidium* decreased across the 3 growth stages by 90.30% (*P* < 0.05), 33.98% (*P* > 0.05), and 44.34% (*P* < 0.05), respectively, compared with CF. Notably, all fertilization treatments increased the abundance of *Fusarium* in the middle and late stages, with increases of 41.19 (*P* > 0.05) and 10.98-fold (*P* < 0.05) relative to F0, and 23.50% (*P* > 0.05) and 2.49-fold (*P* < 0.05) relative to CF, respectively (**Figure 6b**).

### Prediction of soil microbial functions

Bacterial functional profiles were predicted using PICRUSt2 based on the KEGG database. Predicted functions were primarily assigned to six major KEGG categories (**Supplementary Figure 4a**): metabolism, environmental information processing, cellular processes, human diseases, genetic information processing, and organic systems. Among these, metabolism dominated the functional profile, accounting for 74.44% to 76.50% of the total predicted functions across all treatments. Compared with the F0, both SF and CF slightly increased the relative abundance of metabolism-related genes during the flowering and fruiting stage, although the magnitude of change was small (0.06%; **Supplementary Figure 5**). Overall, bacterial functional compositions were highly similar among treatments, indicating that fertilization regimes had only minor effects on the predicted metabolic potential of bacterial communities.

Fungal functional guilds were predicted using FUNGuild. Seven trophic modes were identified in the rhizosphere soil of melon, including Saprotroph, Pathotroph, Symbiotroph, and their combinations (**Supplementary Figure 4b**). Among them (**Supplementary Figure 6**), Saprotrophs were the dominant functional group, with relative abundances ranging from 35.56% to 70.18%. SF treatment significantly enriched saprotrophic fungi at the fruit enlargement and maturity stages, with relative increases of 24.42% and 15.65% compared with CF, respectively. In contrast, Pathotrophs were consistently suppressed under SF, with relative abundances ranging from 0.26% to 10.61%, which were lower than those observed under CF (0.30%–20.20%) and F0 (1.29%–15.32%).

### Correlation analysis among soil microbial communities, soil properties, and melon yield

**Figure 7:**
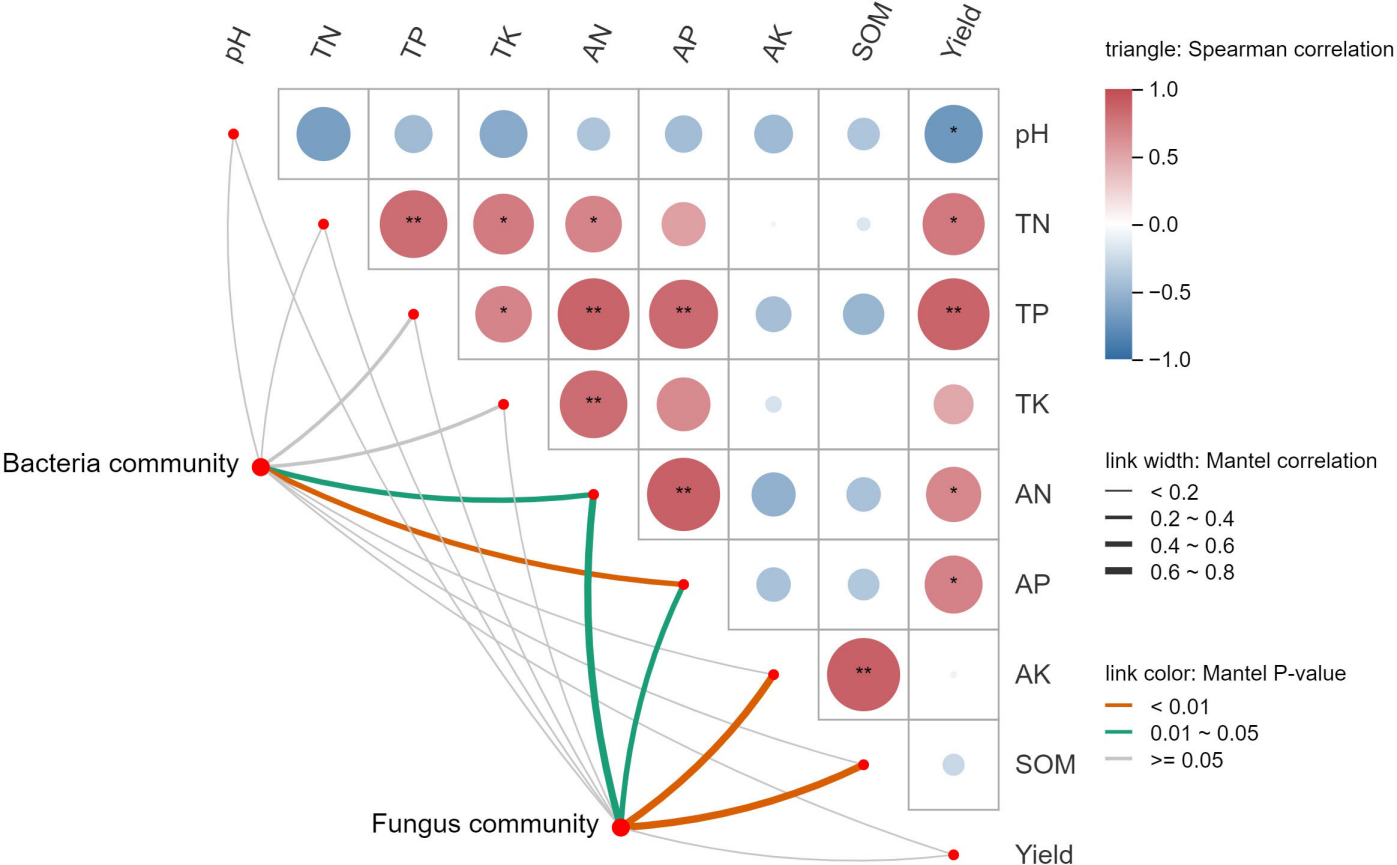
Mantel test analysis of relationships among soil microbial communities, soil physicochemical properties, and melon yield. *P < 0.05; **P < 0.01. TN, total nitrogen; TP, total phosphorus; TK, total potassium; AN, available nitrogen; AP, available phosphorus; AK, available potassium; SOM, soil organic matter.

To elucidate the relationships among soil physicochemical properties, microbial community structure, and melon yield, Mantel tests and redundancy analysis (RDA) were performed. Mantel test results (**Figure 7**) indicated that melon yield was significantly positively correlated with soil available nitrogen (AN), available phosphorus (AP), total nitrogen (TN), and total phosphorus (TP), and significantly negatively correlated with soil pH. These results suggest that soil nitrogen and phosphorus availability are the primary physicochemical drivers of melon yield, while soil pH exerts an important regulatory influence. Furthermore, the correlation between the overall bacterial community structure and AP was significant, whereas extremely significantly observed with AN. The correlations of the whole fungal community with AK and SOM were extremely significant, and only with AN and AP were significant. Notably, neither the bacterial nor the fungal community structure exhibited a significant correlation with melon yield.

**Figure 8:**
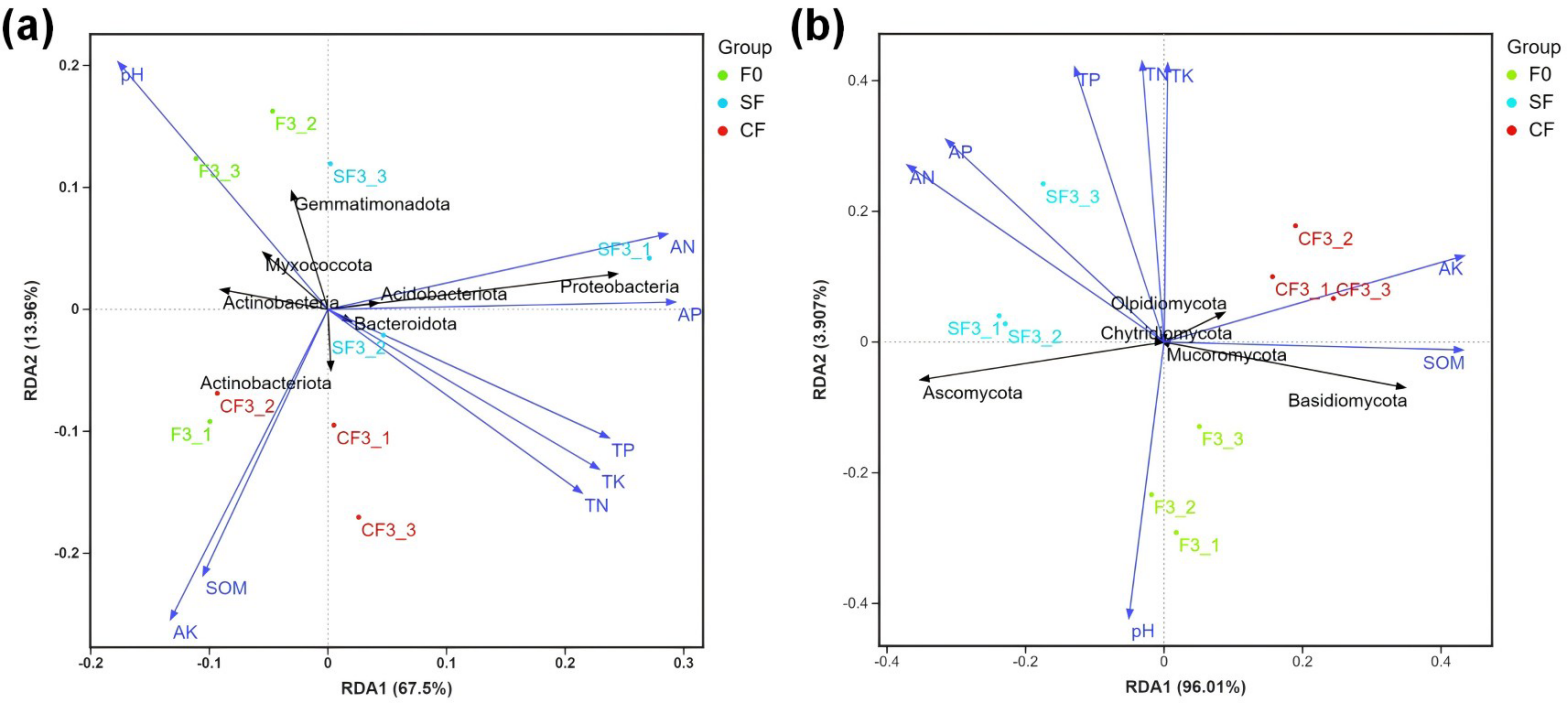
Redundancy analysis (RDA) of soil physicochemical properties and microbial community structure under different fertilization treatments. **(a)** Bacterial communities; **(b)** Fungal communities. Blue arrows represent environmental variables, and black arrows represent microbial phyla. Arrow length indicates the strength of the correlation. Angles < 90° indicate positive correlations, whereas angles > 90° indicate negative correlations. Data are presented for key growth stages: flowering and fruiting (F0-1/CF1/SF1), fruit enlargement (F0-2/CF2/SF2), and maturity (F0-3/CF3/SF3). TN, total nitrogen; TP, total phosphorus; TK, total potassium; AN, available nitrogen; AP, available phosphorus; AK, available potassium; SOM, soil organic matter.

RDA further clarified the relationships between dominant microbial taxa and soil physicochemical properties. For bacteria (**Figure 8a**), the first and second RDA axes explained 67.50% and 13.96% of the total variation, respectively, accounting for 80.46% of the soil bacterial community. Proteobacteria were positively associated with AN and AP, Gemmatimonadota were positively correlated with soil pH, and Actinobacteria was positively correlated with SOM, AK, and pH. For fungi (**Figure 8b**), the first and second axes explained 96.01% and 3.91% of the variation, respectively, accounting for 99.92% of the fungal community structure. Ascomycota was positively correlated with AP, AN, and pH, whereas Basidiomycota were mainly associated with SOM and AK. In conclusion, soil nitrogen and phosphorus availability and soil pH emerged as the key environmental factors directly regulating melon yield. In contrast, soil microbial community composition was primarily shaped by soil physicochemical properties rather than acting as a direct determinant of yield under the conditions of this study.

### Analysis of economic benefits of melon under different fertilization treatments

The economic benefits of melon under different fertilization treatments were analyzed (**Table 3**). The purchase price of melon was estimated at 3 CNY·kg⁻¹. The labor cost for fertilization was 260 CNY·d⁻¹, and one worker could fertilize an area of 10 × 667 m² per day. The prices of urea, phosphorus, potassium sulfate, and slow-release fertilizer were 3.5, 4.0, 3.3, and 3.5 CNY·kg⁻¹, respectively. SF treatment adopted one-time basal application, whereas CF required approximately four additional topdressings. Except for fertilization, all other field expenditures were kept consistent between the two treatments. Compared with CF, SF treatment increased the economic benefit of melon by 3826.36 CNY·hm⁻². In summary, SF treatment not only substantially reduced labor input for fertilization but also increased the net economic benefit.

**Table 3:** Effects of different fertilization treatments on economic benefits of melon.

Treatment	Output value/CNY·hm⁻2	Fertilizer cost/CNY·hm⁻2	Labor cost/CNY·hm⁻2	Economic benefit/CNY·hm⁻2
SF	143,820	3,191	390	140,239
CF	142,830	4,468	1,950	136,412

CF indicates conventional fertilization, and SF indicates deep application of slow-release fertilizer at a depth of 35 cm. Economic benefit (CNY·hm⁻2) =Output value - Fertilizer cost - Labor cost (assuming other costs are equivalent across treatments).

## Discussion

Xinjiang is one of the major melon-producing regions in China, characterized by extensive cultivation areas, high yield, and superior fruit quality, and it plays a critical role in the regional agricultural economy (Zhu et al., 2021). In the context of the current low-carbon economy, the use of slow-release fertilizers to reduce costs and improve nutrient-use efficiency is of great importance for the sustainable development of agriculture (Li et al., 2021a). However, information remains limited regarding how the application depth of slow-release fertilizer influences melon nutrient uptake and yield in this region. Our results demonstrate that the accumulation and distribution of nitrogen (N), phosphorus (P) and potassium (K) in melon under different fertilization management had obvious regularity during growth period, that is, nitrogen is predominantly accumulated in leaves at the flowering and fruiting and fruit enlargement stage, then remobilized to fruits at the maturity stage. By contrast, phosphorus and potassium mainly accumulate in leaves during the flowering and fruiting stage and are continuously translocated to fruits in the fruit enlargement and maturity stages. This pattern is consistent with previous reports describing nutrient accumulation dynamics in melon cultivation (Cabello et al., 2011). It can be concluded that moderate nitrogen supply should be maintained from flowering and fruiting to fruit enlargement stage to support nutrient accumulation in leaves. Phosphorus and potassium should be supplied in advance at the flowering stage, whereas from fruit enlargement to maturity stage, nitrogen application should be strictly controlled while phosphorus and potassium are continuously supplied. In the maturity stage, the accumulation of nitrogen and phosphorus in fruits treated with SF35 was higher than that of CF, and the accumulation of potassium was slightly lower than that of CF. The differences between the two treatments were not significant. These patterns closely reflect the nutrient supply characteristics of the respective fertilization regimes: deep-applied slow-release fertilizer primarily enhances soil N and P availability, whereas CF preferentially increases available soil K. Notably, these nutrient accumulation profiles were well aligned with observed fruit quality traits. Among the treatments, deep application of slow-release fertilizer at 35 cm (SF35) resulted in the highest overall yield, single-fruit weight, and soluble solid content, while CF produced fruit with the highest vitamin C content. These differences can be explained by the distinct physiological roles of mineral nutrients: nitrogen primarily promotes crop yield, phosphorus contributes to the development of ester-related aroma compounds, and potassium plays a key role in enhancing vitamin C accumulation in fruits.

This study also showed that the yield under the SF35 treatment was only 0.6% higher than under CF, which was considerably lower than the yield gains reported for slow-release fertilizers in many other agricultural systems. This relatively limited yield response may be attributed primarily to severe drought stress, which is a key factor constraining fertilizer efficiency (Lei et al., 2024; Sulaman et al., 2025). In addition, the predominant soil type in this area for melon production is irrigated brown desert soil, characterized by weak water and nutrient retention capacity and low organic matter content (Liu et al., 2026). Under such conditions, simple substitution of fertilizer type is unlikely to produce substantial yield increases in the short term (Lazcano et al., 2013). Nevertheless, this difference remains meaningful from a practical production perspective. Deep application of slow-release fertilizer allows for one-time fertilization, eliminating the need for repeated topdressing during the growing season. This substantially simplifies field operations, reduces labor costs, and aligns with the trend toward large-scale and labor-efficient agricultural system (Sim et al., 2025). Moreover, the stable nutrient release, high nutrient-use efficiency, and low environmental risk associated with slow-release fertilizers help mitigate problems such as nutrient imbalance, fertilizer loss, and environmental pollution (Chen et al., 2022). Over the long term, this approach supports both yield stability and production efficiency. In conclusion, deep application of slow-release fertilizer represents a highly effective and simplified fertilization strategy for melon cultivation in Xinjiang region, offering clear agronomic and environmental advantages and strong potential for wider adoption.

Fertilization significantly affects the structure of soil microbial communities, influencing nutrient availability as well as microbially mediated processes related to plant growth and physiological regulation (Guo et al., 2020). In this study, SF consistently increased the ACE index of root-associated bacteria throughout the entire growth stage, in agreement with previous studies (Chen et al., 2022; Meng et al., 2024b). At the phylum level, SF increased the abundance of Proteobacteria during the middle and late growth stages. Studies have shown that Proteobacteria is widely recognized for its ability in phosphorus solubilization and organic matter decomposition (Fierer et al., 2012). At the genus level, SF predominantly enriched *Rhodanobacter* and *Devosia* in the rhizosphere. Species within *Rhodanobacter* are known to possess denitrification functions and play an important role in the soil nitrogen cycling by reducing nitrate (Zhou et al., 2017). *Devosia* has also been reported as a nitrogen-fixing bacterial genus, contributing to soil nitrogen inputs and plant nutrient acquisition (Rivas et al., 2002). In contrast, CF mainly increased the abundance of *Gemmatimonas*, *Pseudolabrys*, and *Sphingomonas*. *Gemmatimonas* is primarily involved in the decomposition and transformation of complex organic carbon in the soil through secreting various hydrolases to degrade cellulose, hemicellulose, and other polysaccharide substances in plant residues, thereby contributing to soil carbon cycling (Bao et al., 2025). *Pseudolabrys*, originally isolated from alpine soils, exhibits strong tolerance to low temperatures (Donhauser et al., 2020), and may function as a stress- adaptive bacterium that enhances plant resilience. *Sphingomonas* species have been reported to alleviate cadmium toxicity, produce carotenoids, and improve stress tolerance of rice (Cheng et al., 2021). In summary, SF preferentially promotes bacterial taxa involved in nitrogen cycling, while CF enriches beneficial bacterial communities involved in carbon cycling and plant stress tolerance. In the fungal community, all fertilization treatments reduced the richness and diversity of root-associated fungi. This pattern is consistent with previous findings showing that single fertilizer application can induce soil acidification, thereby suppressing fungal development (Qu et al., 2025). Additionally, this trend reflects the natural vertical distribution of fungal communities, which typically exhibit peak richness and diversity in surface soils (0–30 cm) and decline markedly with increasing soil depth (Guo et al., 2025). Across all treatments, Ascomycota was the dominant fungal phylum. Compared to CF, SF significantly increased the abundance of Ascomycota during the middle and late growth stages. Most Ascomycota are saprophytic fungi, capable of decomposing recalcitrant organic compounds such as lignin and keratin, thereby playing an important role in nutrient cycling (Yelle et al., 2008). Moreover, Ascomycota are generally characterized by an r-strategy life history, and their abundance tends to increase under nutrient-rich conditions (Guo et al., 2020). The Olpidiomycota phylum, which is often considered sensitive to changes in microbial diversity (Li et al., 2019), showed reduced abundance under SF throughout the whole melon growth period compared with CF. *Olpidium* species are usually recognized as plant pathogens (Stanghellini et al., 2010) or virus vectors (Ohki et al., 2010) and are associated with growth inhibition and disease incidence in crops (Windisch et al., 2021). Although some studies have suggested potential beneficial roles of *Olpidium* (Yang et al., 2023), evidence supporting such effects remains limited, whereas its detrimental impacts are more consistently documented. We also found that SF significantly increased the abundance of *Penicillium* during the middle and late stages of melon fruit development. *Penicillium* species are well known for their ability to solubilize inorganic phosphorus and convert it into plant-available forms, thereby enhancing phosphorus utilization efficiency (Suraby et al., 2023). Notably, all fertilization treatments significantly increased the abundance of *Fusarium* in rhizosphere soil during the middle and late growth stages. This observation is consistent with previous reports indicating that inorganic fertilizer application tends to increase the relative abundance of potential pathogenic fungi, while organic amendments can suppress their proliferation (Hu et al., 2017). Overall, these findings provide important insights for optimizing fertilization management strategies, highlighting the role of fertilizer type and application depth in shaping rhizosphere microbial communities and their associated nutrient cycling functions.

Long-term fertilization is recognized as an effective strategy for regulating crop yields, as it alters soil physicochemical properties and rhizosphere microbial community structure, thereby influencing crop growth, development and yield formation (Zhong et al., 2020; Wang et al., 2021b). Correlation analysis revealed that melon yield was significantly positively correlated with total nitrogen (TN), total phosphorus (TP), available nitrogen (AN), and available phosphorus (AP), while exhibiting a significant negative correlation with soil pH. In contrast, the bacterial and fungal community structures were primarily regulated by AP, available potassium (AK), soil organic matter (SOM), and soil pH. Soil pH is a core indicator of soil physicochemical status and plays a critical role in shaping crop growth environments. Regulating soil pH to optimize rhizosphere conditions is therefore a key technical approach for improving crop productivity in agricultural systems (Lammel et al., 2018; Rousk et al., 2010). Previous studies have clearly defined the optimal pH range for melon growth as 6.0-7.0 (Alfareza and Erlina, 2023). In this study, the soil pH under the SF treatment was between 6.07-6.62, falling squarely within this optimal range. This finding indicates that SF can effectively regulate soil acidity and alkalinity, thereby creating favorable conditions for melon growth and yield enhancement. Xinjiang is located in the arid region of northwest China, where farmland soils are typically alkaline (Wei et al., 2016). Such alkaline conditions impose strong constraints on nutrient availability. High concentrations of calcium, magnesium and other base cations in alkaline soils promote ammonia volatilization, leading to nitrogen loss (Fenn and Kissel, 1973), while readily available phosphorus tends to react with calcium and magnesium to form insoluble phosphate compounds that are poorly accessible to plant roots (Liu et al., 2021). Consequently, the bioavailability of nitrogen and phosphorus is substantially reduced under alkaline soil conditions. In this study, the deep application of slow-release fertilizer enabled the sustained release of nitrogen and phosphorus in the soil, effectively alleviating these limitations. This fertilization strategy therefore addresses key nutrient constraints in alkaline soils and highlights the suitability and practical value of deep-applied slow-release fertilizers for melon production in Xinjiang.

This study also found no significant correlation between the overall structure of the microbial community and melon yield, which is consistent with the findings of previous research in a closed hydroponic system (Lin et al., 2021). This finding suggests that the explanatory power of overall microbial community structure for crop yield may be limited. Collectively, these findings indicate that crop yield is not directly and simply determined by the overall microbial community structure. Instead, environmental factors (e.g., water and nutrient availability) and specific functional microbial groups are likely to play more direct and dominant roles in regulating crop productivity (Zhongyou et al., 2017). Overall, these results demonstrate that soil nitrogen and phosphorus availability, together with soil pH, are the primary environmental factors directly regulating melon yield. Although bacterial and fungal community structures were strongly influenced by soil physicochemical properties such as AN, AP, AK, SOM and pH, they did not exert a direct effect on melon yield under the conditions of this study. These findings provide a theoretical basis for clarifying the coupling relationships among soil environment, microbial community, and melon yield under different fertilization management regimes.

## Conclusions

From a soil–microbe–plant perspective, this study investigated the effects of deep application of slow-release fertilizer in melon production in Xinjiang. Deep application of fertilizer at 35 cm can enhance soil nitrogen and phosphorus supply, enrich functional beneficial microbial communities, increase rhizosphere bacterial diversity, and adjust soil pH to the optimal range for melon growth. Soil nitrogen and phosphorus contents and pH are direct key factors regulating melon yield. Although rhizosphere microorganisms did not directly dominate yield in the short term, they may indirectly influence yield formation by driving nutrient transformation. As this study was conducted over a single growing season, the long-term effects of this fertilization method on soil microecology and productivity still require verification through multi-year field experiments. Nevertheless, our findings confirm that a single deep application can achieve a stable yield of melon while reducing labor costs and improving economic benefits. Collectively, these findings provide a solid theoretical basis for the development and optimization of simplified, labor-efficient fertilization systems for melon cultivation in Xinjiang and other arid regions of northwest China.

## Data Availability

The datasets presented in this study can be found in online repositories. The names of the repository/repositories and accession number(s) can be found below: https://www.ncbi.nlm.nih.gov/, PRJNA1404602 https://www.ncbi.nlm.nih.gov/, PRJNA1404617.
