# Supplementary material for: Effects of deep slow-release fertilizer on melon yield, economic benefits and rhizosphere microorganisms
Source: Front Plant Sci. 2026 Jun 3;17:1831940. doi: 10.3389/fpls.2026.1831940 (PMC13272342; doi:10.3389/fpls.2026.1831940)
Supplement: Supplementary file 1 [file DataSheet1.docx]

**Supplementary Files**


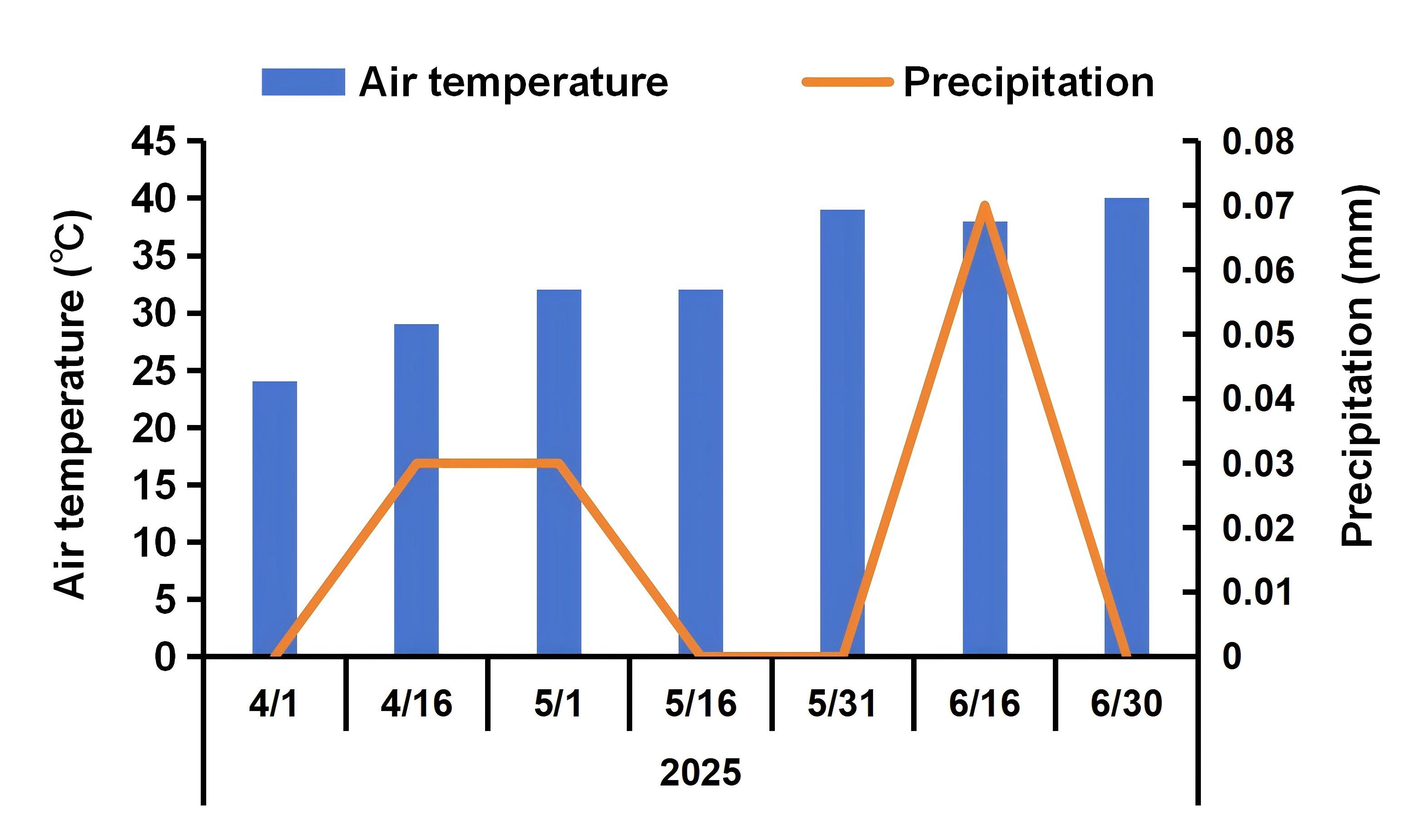


Figure S1. Precipitation and air temperature during the melon growing season in 2025.


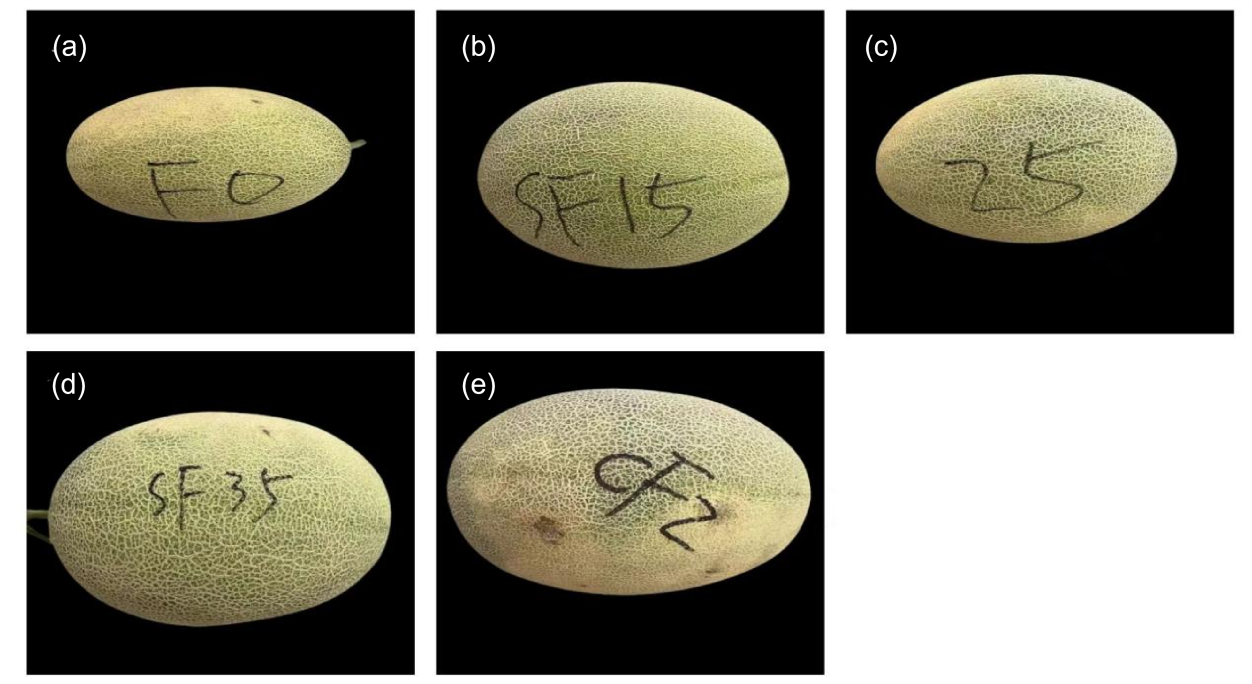


Figure S2. Fruit appearance of melon under different fertilization treatments. (a) F0 indicates no fertilization; (b) SF15 indicates deep application of slow-release fertilizer at a depth of 15 cm; (c) SF25 indicates deep application of slow-release fertilizer at a depth of 25 cm; (d) SF35 indicates deep application of slow-release fertilizer at a depth of 35 cm; (e) CF indicates conventional fertilization.


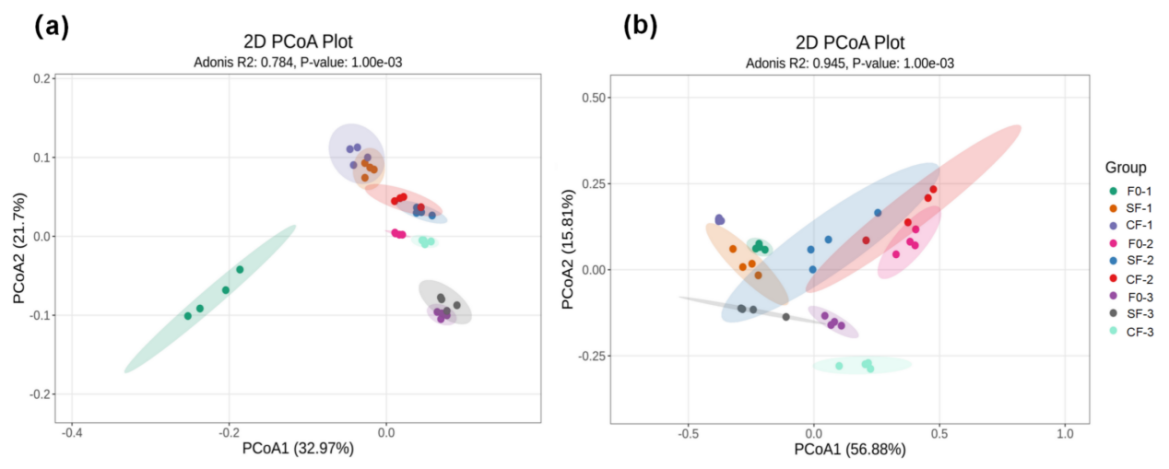


Figure S3. The impact of different fertilization treatments on bacterial (a) and fungal (b) β-diversity. F0-1, CF1, and SF1 correspond to the flowering and fruiting stage; F0-2, CF2, and SF2 correspond to the fruit enlargement stage; and F0-3, CF3, and SF3 correspond to the maturity stage. F0 indicates no fertilization, CF indicates conventional fertilization, and SF indicates deep application of slow-release fertilizer at a depth of 35 cm.


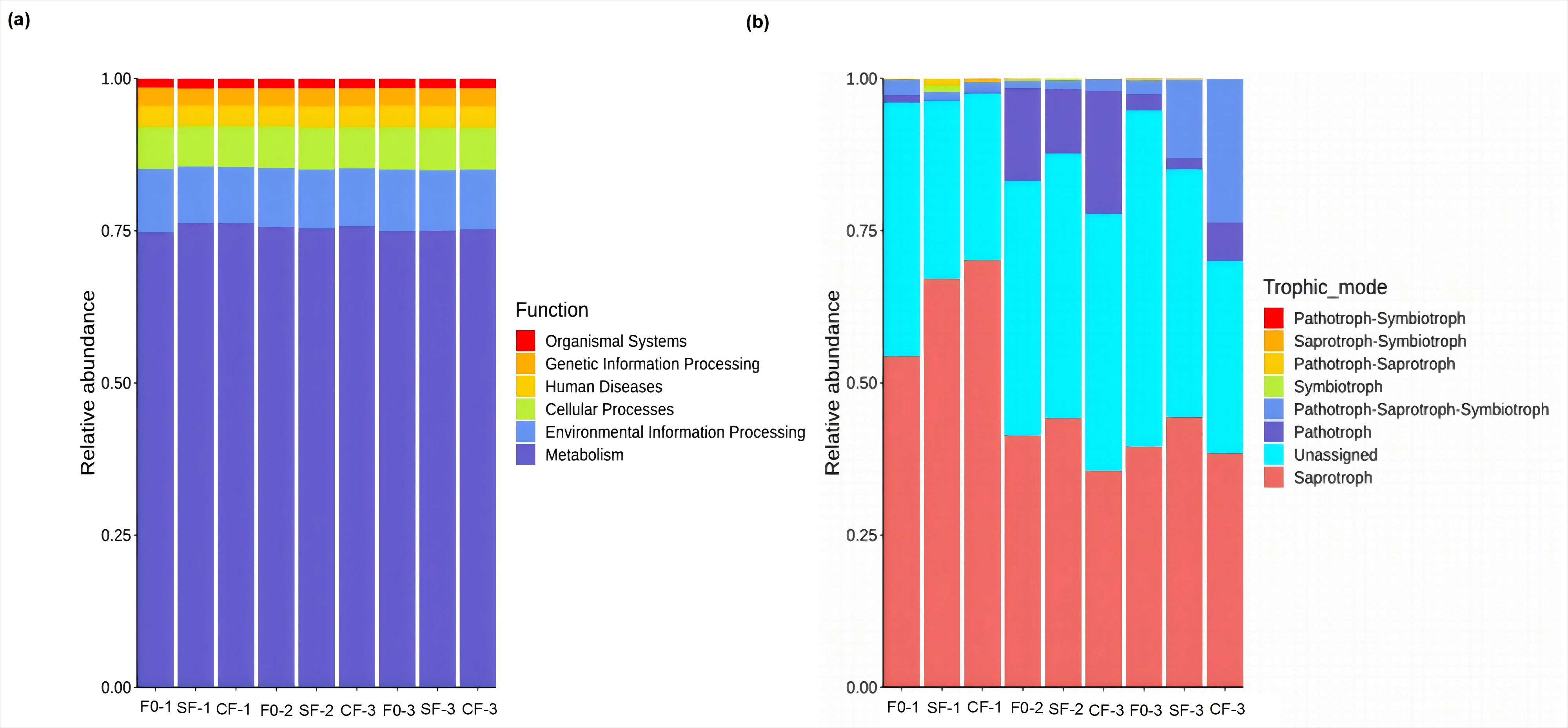


Figure S4. Relative abundance of predicted functional categories of soil microbial communities.

(a) Bacterial communities predicted by PICRUSt2 based on the KEGG database; (b) Fungal communities predicted by FUNGuild. SF indicates deep application of slow-release fertilizer at 35 cm; CF indicates conventional fertilization; F0 indicates no fertilization. Data are presented for key growth stages: flowering and fruiting (F0-1/CF1/SF1), fruit enlargement (F0-2/CF2/SF2), and maturity (F0-3/CF3/SF3).


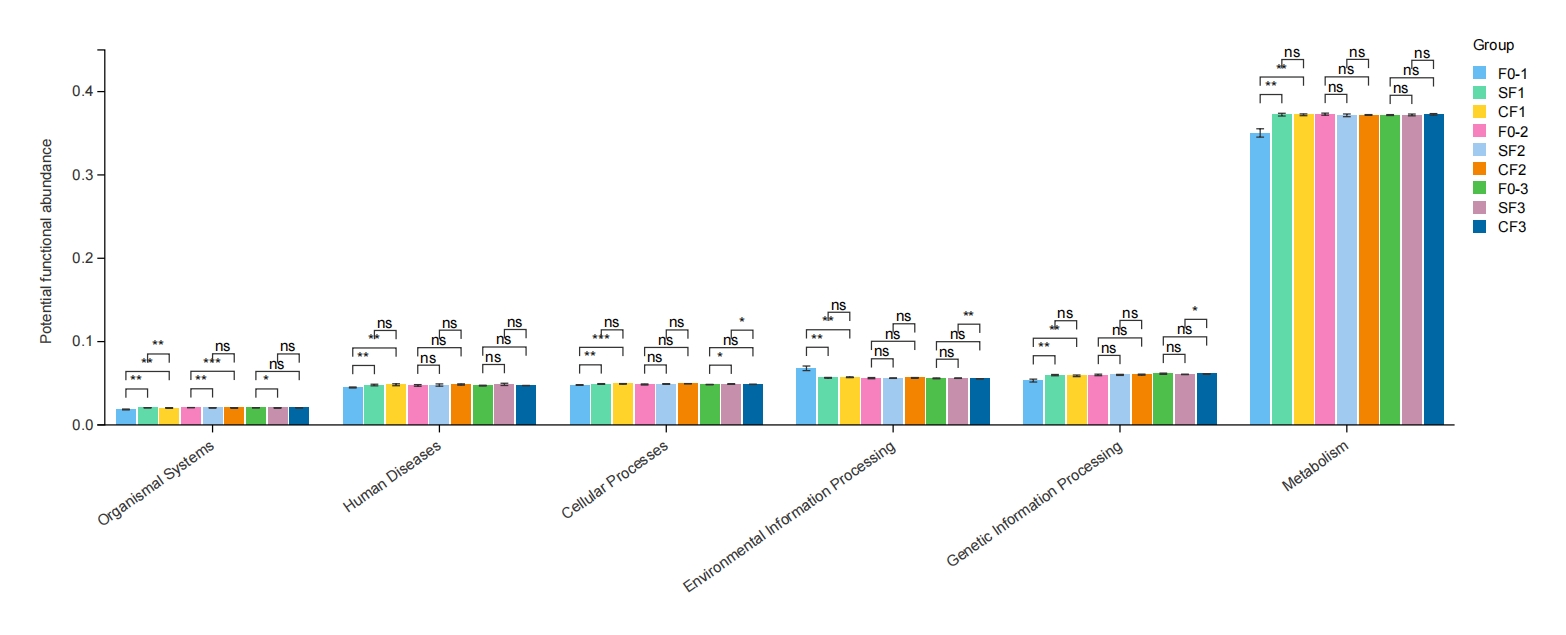


Figure S5. Predicted first-level KEGG functional pathways of bacterial communities. SF indicates deep application of slow-release fertilizer at 35 cm; CF indicates conventional fertilization; F0 indicates no fertilization. Data are presented for key growth stages: flowering and fruiting (F0-1/CF1/SF1), fruit enlargement (F0-2/CF2/SF2), and maturity (F0-3/CF3/SF3).


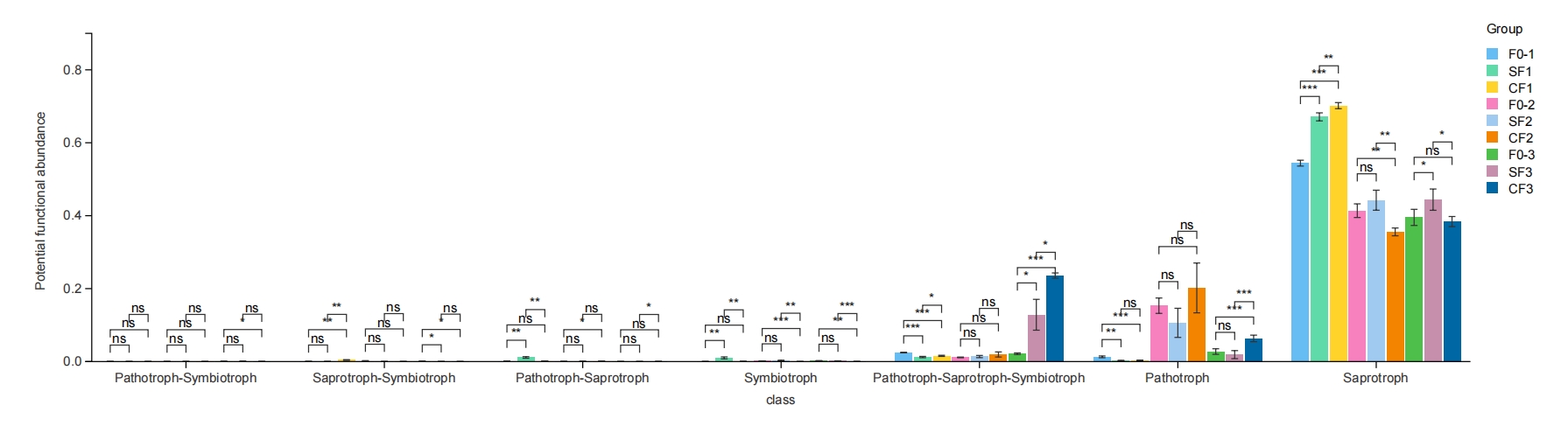


Figure S6. Predicted trophic modes (functional guilds) of fungal communities. SF indicates deep application of slow-release fertilizer at 35 cm; CF indicates conventional fertilization; F0 indicates no fertilization. Data are presented for key growth stages: flowering and fruiting (F0-1/CF1/SF1), fruit enlargement (F0-2/CF2/SF2), and maturity (F0-3/CF3/SF3).
